# IL-10 secreted by M2 macrophage promoted tumorigenesis through interaction with JAK2 in glioma

**DOI:** 10.18632/oncotarget.12317

**Published:** 2016-09-28

**Authors:** Ling Qi, Hongquan Yu, Yu Zhang, Donghai Zhao, Peng Lv, Yue Zhong, Ye Xu

**Affiliations:** ^1^ The Department of Pathology, Jilin Medical University, Jilin 132013, PR China; ^2^ The Department of Neurosurgery, First Affiliated Hospital of Jilin University, Jilin 130021, PR China; ^3^ The Department of Science and Technology, Jilin Medical University, Jilin 132013, PR China; ^4^ The Medical Research Laboratory, Jilin Medical University, Jilin 132013, PR China

**Keywords:** glioma, M2 macrophage, proliferation, IL-10, JAK2/STAT3

## Abstract

M2 tumor-associated macrophage has been found to play a supportive role in the progression of glioma. The underlying mechanism, nevertheless, has been largely unknown. In our study, to investigate how M2 macrophage played role in glioma, firstly we've analyzed the clinicopathological significance of M2 macrophage existence on clinical tissues of glioma using detection of CD163 expression with immunohistochemistry. Then, we've artificially induced M2 macrophage from human monocyte cell line THP-1, followed by co-culture with glioma cell lines *in vitro*. It was found that M2 macrophage was shown to be markedly distributed in glioma relative to paired normal control; and high prevalence of M2 macrophage was significantly associated with poorer overall survival and tumor progression. Moreover, M2 macrophage was found to be able to promote the growth *in vitro* and tumorigenesis *in vivo* in xenografted mice model. Mechanistically, it is IL-10 from M2 macrophage that was shown to promote proliferation, dependent on activation of JAK2/STAT3 pathway. Further, IL-10 was found to be able to interact with JAK2 in glioma cells. Taking together, we for the first time found that IL-10 from M2 macrophage promoted proliferation of glioma through interaction with JAK2; thereby activating the JAK2/STAT3 pathway, indicative of IL-10 could be used as a therapeutic target in the curing of glioma.

## INTRODUCTION

Glioma is aggressive, rapidly progressing, infiltrative, parenchymal neoplasm [1], with median survival being less than 16 months in spite of optimal treatment. Advances in surgical, radiation, and conventional chemotherapies have had hardly impact on the prognosis of the aggressive disease [2]. The refractory glioma to standard therapies is regarded to spring from phenotypic heterogeneity and diffuse infiltration into normal brain parenchyma as well as residence within the unique immune environment of the central nervous system [3]. In light of these clinical changes and the immune environment of glioma, immunotherapy is becoming increasingly appealing treatment for glioma [4].

Macrophages, specifically referred to as tumor-associated macrophages (TAMs), are the most common cell types found among tumor-infiltrating immune cells [5]. Although abundant macrophage infiltration is a common feature of glioma, these TAMs lack apparent phagocytic activity [3]. Instead, TAMs from glioma secreted or expressed some cytokines, such as IL-10, transforming growth factor beta (TGF-β), and other solute factors [6]. These cytokines were discovered not only to reduce the anti-tumor activity of T cells and natural killer cells but also to promote the tumor proliferation, infiltration and angiogenesis.

TAMs can be categorized into M1 and M2 subtypes on the basis of their polarization status [2]. In tumors in effect, the M1 or M2 subtypes TAMs stands for tumor suppressive or supportive, respectively [7]. Several cell surface markers, such as CD68, CD163 and CD204 have been employed to mark M2 subtype, while iNOS, CD11c and MHCII have been proposed for M1 subtypes. It has been found that TAMs in astrocytomas with lower grade were strongly stained with the M1 marker MHCII, whereas manifested strongly with M2 marker staining in glioma [8]. Such findings suggest that M2 type TAMs might play a pivotal role in the progression of glioma. However, the underlying mechanism by which M2 macrophage involves in glioma remains unknown.

In the setting, to investigate the possible mechanism by which M2 type TAMs played in glioma, we've artificially induced M2 type macrophage from human monocytes that are commercially available; apart from phenotypic correlation analysis performed between M2 type macrophage and clinicopatholgical characteristics of glioma on clinical tissues. Then it was subjected to co-culture with glioma cell lines *in vitro*. It was found that M2 type macrophage co-cultured with glioma cell lines was capable of promoting cell gowth *in vitro* and tumorigenesis *in vivo* in nude mice model. Mechanistically, we found that it is IL-10 from M2 type macrophage that was displayed to promote growth through activation of JAK/STAT signaling pathway in glioma cells *in vitro*.

## RESULTS

### M2 macrophages were pronouncedly existed in glioma relative to paired normal control

**Figure 1:**
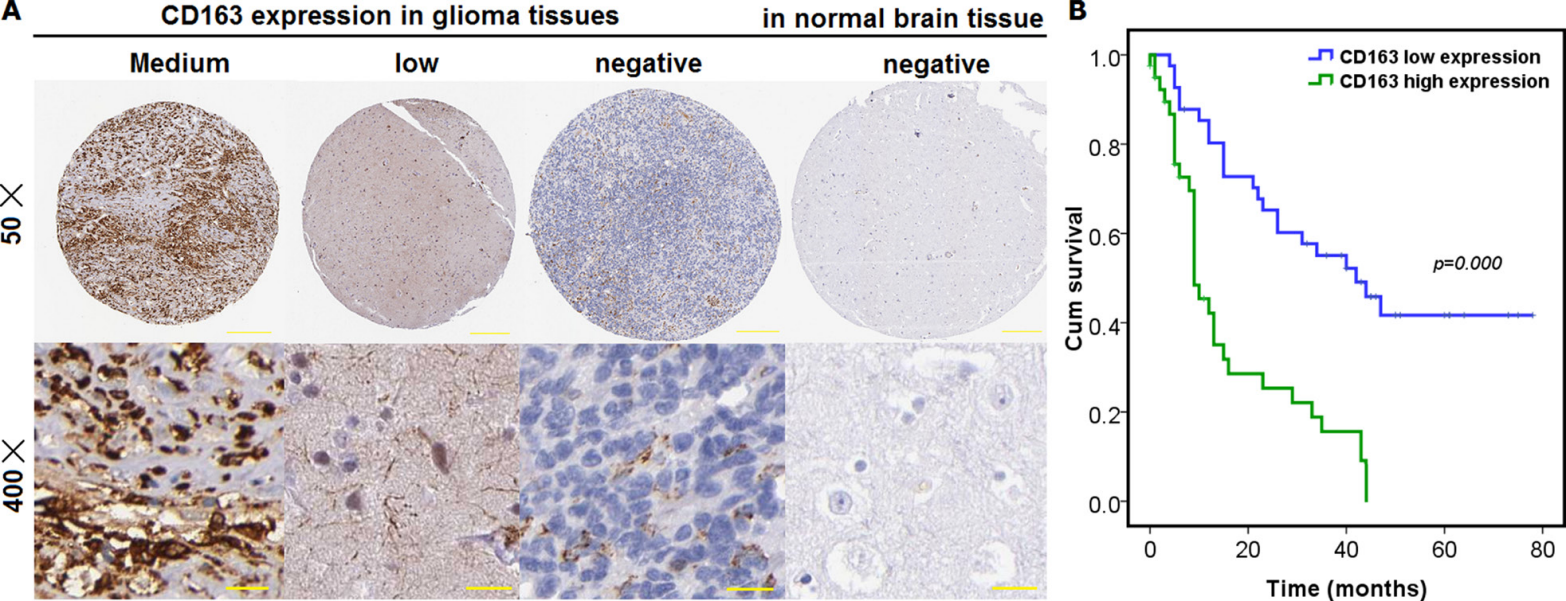
Existence and expression of M2 type macrophage was detected and its prognostic significance was also analyzed in glioma tissues, totaling 90 cases, as well as in paired normal control tissue (**A**) M2 macrophage was evaluated using IHC with CD163, a commonly accepted specific marker for M2 type macrophage. The immunostaining of CD163 was cytoplasmic and membranous, with its expression being medium, low and negative in glioma tissues and normal brain tissues. Scale bar denotes 50 μm; (**B**) overall prognostic significance was analyzed using Kaplan-Meier survival curve wherein Log-rank test was employed.

M2 type macrophage has been reported to be clinically relevant with progression of glioma [8]. To confirm the association between existence of M2 macrophages and clinicopathological variables, we've firstly detected the existence of M2 macrophages in glioma tissues and paired normal controls in our setting, using IHC method with the cell surface marker CD68 (pan-macrophage marker) in combination with CD163 (M2 macrophage marker), which have been widely adopted and employed in the evaluation of M2 macrophage prevalence in cancers. Positive Immunostaining of CD163 was shown to be remarkably distributed in glioma tissues in comparison with paired normal controls (Figure 1A). That is, the number of M2 macrophages infiltrated in glioma tissues was significantly more than that in paired normal controls. Meanwhile, we've also detected the CD68, a well-accepted pan-macrophage marker, using IHC method. CD68 was also shown to be significantly existed in glioma tissues versus paired normal controls (Supplementary Figure S1). To further confirm the observation we've made from tissue level, we've also detected the M2 macrophages in peripheral blood sampled from patients with glioma as well as from normal healthy controls, by means of evaluation of the ratio of macrophage positive for CD168, M2 macrophage marker, to leukocyte positive for CD45, pan-leukocyte marker (Supplementary Figure S2A). It was shown that the ratio of CD168+/CD45+ was markedly higher in peripheral blood from patients with glioma than that in healthy controls (Supplementary Figure S2C). These data we obtained indicate that M2 macrophages were tumor-promoting in glioma tissues compared with normal controls.

### Existence of M2 macrophages was shown to be markedly associated with tumor progression and poorer overall prognosis of glioma

To understand the clinical relevance of M2 macrophage observed to be extensively infiltrated into glioma tissues, correlation was analyzed using Cross-table statistical approach between the expression of CD163 and clinicopathological variables. M2 macrophages were shown to be remarkably associated with world health organization (WHO) grade, suggesting that M2 type macrophage was clinically and significantly relevant to the progression of glioma (Table 1). In addition to the analysis of clinicopathological significance of M2 macrophage, we've also analyzed the prognostic significance of M2 macrophage existence in glioma. We found that there was pronounced association between M2 macrophage and overall prognosis (Figure 1B), suggesting that the higher the M2 macrophage was, the poorer the overall prognosis will be. To further evaluate the effect exerted over the overall prognosis of glioma by M2 macrophages, both Univariate and Multivariate Cox regression analysis were performed. It was found that M2 subtype macrophage was an independent prognostic factor for patients with glioma (Table 2).

**Table 1:** Analyzed was clinicopathological significance of CD163 expression in glioma tissues

Variable	group	Total	CD163 expression		Chi-square	*p* value
			Low (−, +)	High (++)		
Glioma		140	49	91	50.473	0.000
Paired normal control		140	108	32		
Age	< 60	67	23	44	0.025	1.000
	≥ 60	73	26	47		
WHO grade						
	I + II	60	26	34	3.205	0.050
	III + IV	80	23	57		
Gender						
	female	43	13	30	0.620	0.451
	male	97	36	61		
Relapse						
	Yes	105	29	76	10.058	0.002
	No	35	20	15		
Histological subtype						
	Astrocytic	49	20	29	1.143	0.565
	Oligoastrocytic	45	14	31		
	Oligodendroglial	46	15	31		

**Table 2:** Univariate and multivariate Cox-regression analysis of the CD163 expression over prognostic parameters in patients with glioma

Clinicopathological parameters	Univariate analysis		Multivariate analysis	
	RR (95% CI)	*p*	RR (95% CI)	*p*
Age (*n* = 140)	1.192 (0.582, 2.44)	0.632		
Relapse (*n* = 140)	2.652 (1.254, 5.606)	0.011	3.083 (1.252, 7.595)	0.014
WHO Grade (*n* =140)	2.651 (0.988, 7.116)	0.053	2.661 (0.875, 8.092)	0.085
Gender (*n* = 140)	1.073 (0.476, 2.418)	0.865		
Histological subtype (*n* = 140)	0.930 (0.418, 2.072)	0.859		
CD163 immunostaining score	2.141 (1, 4.582)	0.05	2.447 (1.014, 5.907)	0.047

### M2 macrophages were displayed to be able to promote proliferation, migration and invasion of glioma cells *in vitro*

**Figure 2:**
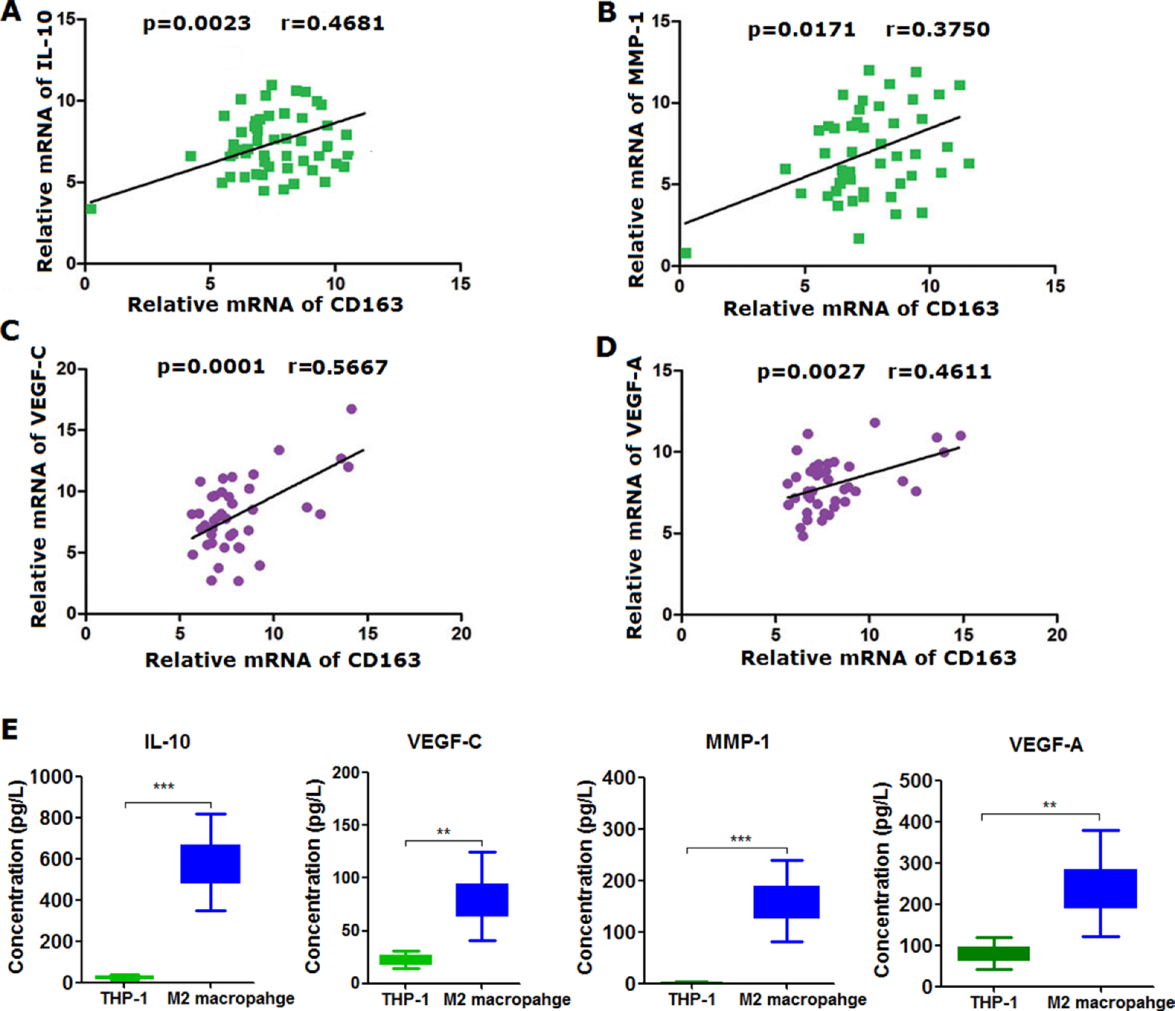
IL-10, VEGF-A, VEGF-C and MMP-1 were markedly secreted by M2 macrophage induced from THP-1 as compared with THP-1 cells (**A**–**D**) correlation was analyzed using Pearson Correlation analysis method between expression of IL-10, VEGF-A, VEGF-C and MMP-1 and CD163 on mRNA level in M2 macrophages induced from THP-1; (**E**) IL-10, VEGF-A, VEGF-C and MMP-1 secreted from M2 and THP-1 was evaluated using ELISA method in the culture media of M2 macrophage and THP-1 cells, respectively. Two-tailed Independent sample *T* test was employed, * stands for *p* < 0.05, ** stands for *p* < 0.01 and *** represents *p* < 0.001 in comparison with control group.

**Figure 3:**
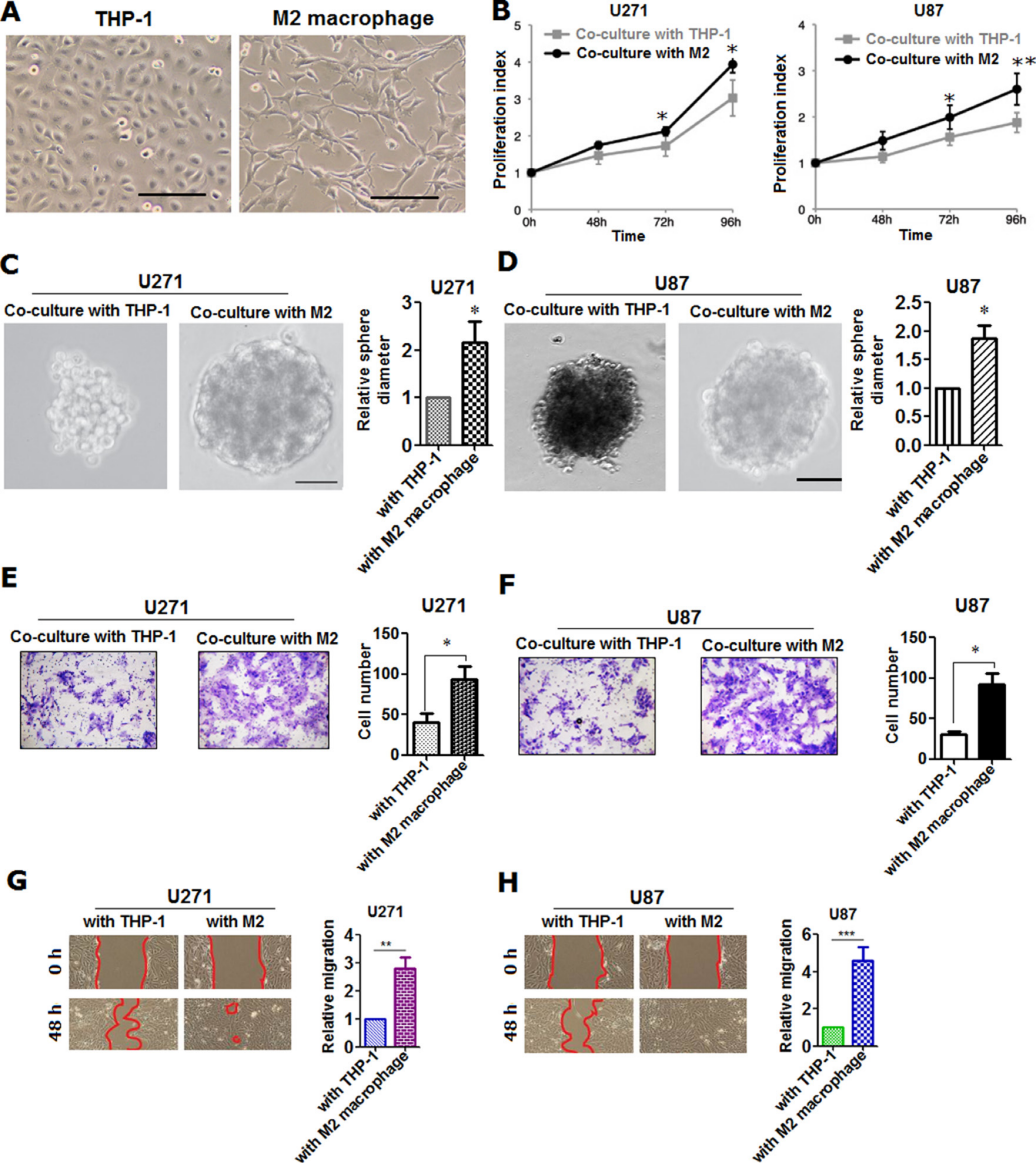
M2 macrophage was shown to be able to promote the proliferation, invasion and migration of glioma cells (**A**) morphological phenotype was presented using Phase contrast microscopy of THP-1 and M2 macrophage, induced from THP-1 cell as recommended by previous studies. Scale bar represents 100 μm; (**B**) proliferative variation was detected using MTT method in glioma cells U271 and U87, co-cultured with M2 macrophage cells induced from THP-1 and with the same number of THP-1 cells treated with M-CSF, used as control; (**C–D**) spheroid phenotype of glioma cells was captured using Phase contrast microscope in U271 and U87 co-cultured with THP-1 and M2 macrophage induced. Presented was representative spheroid that had detached from the monolayer and was floating freely in the culture media. Shown was both qualitative and quantitative spheroid, Scale bar was 25 μm; (**E**) invasive variation was qualitatively and quantitatively evaluated using Transwell assay in U271 cells co-cultured with THP-1 and M2 macrophage; (**F**) in parallel, invasive assay was performed in U87 glioma cells as U271 was did; (**G–H**) migratory variation was qualitatively and quantitatively evaluated using wound-healing assay in U271 and U87 glioma cells co-cultured with THP-1 and M2 macrophage, respectively. All the detection was carried out independently in triplicate, and representative figures were presented here. Two-tailed Independent sample T test was employed, * represents *p* < 0.05, ** represents *p* < 0.01 and *** means *p* < 0.001 compared with control group.

Having understood the clinical relevance of M2 macrophage infiltrated in glioma tissues, we've next continued to explore the role of M2 macrophage in glioma cells *in vitro*. To begin with, we've artificially induced M2 type macrophage from THP-1, monocytes from human peripheral blood in accordance with the previously published report [9]. M2 macrophage was identified and sorted using flow cytometry with detection of markers of both CD68 and CD163 after being induced (Supplementary Figure S2B). Results of flow cytometry confirmed that more than 95% of THP-1 were successfully differentiated into M2 macrophages under the exposure to monocyte colony stimulating factor (M-CSF), IL-4, IL-10 and IL-13 (Supplementary Figure S2D). To further verify, we've detected some cytokines closely related to M2 macrophage on mRNA level using qRT-PCR, including IL-10, VEGF-A, VEGF-C, MMP-1 and CD163 (Supplementary Figure S3A). In addition, we've also measured the secreted cytokines we've selected using ELISA approach (Figure 2E). It can be seen that both of these cytokines and chemokines were all significantly higher in M2 macrophage relative to monocytes (Figure 2A–2D), indicating that M2 macrophage was induced with success (Figure 3A). Based on which, we've co-cultured glioma cell lines U271 and U87 with M2 macrophage we've induced (Supplementary Figure S3B). Subsequently, we've evaluated the proliferative, migratory and invasive variation of U271 and U87 cells co-cultured with or without M2 macrophages. It was discovered that M2 macrophage was remarkably able to promote proliferation (Figure 3B–3D), invasion (Figure 3E–3F) and migration (Figure 3G–3H) of glioma cells U271 and U87 in comparison with co-culture with THP-1 treated with macrophage colony stimulating factor (M-CSF), indicating that M2 macrophages induced from monocytes were capable of promoting proliferation, invasion and migration of glioma cells *in vitro*.

### M2 macrophages promoted tumorigenesis *in vivo* in xenografted mice model

**Figure 4:**
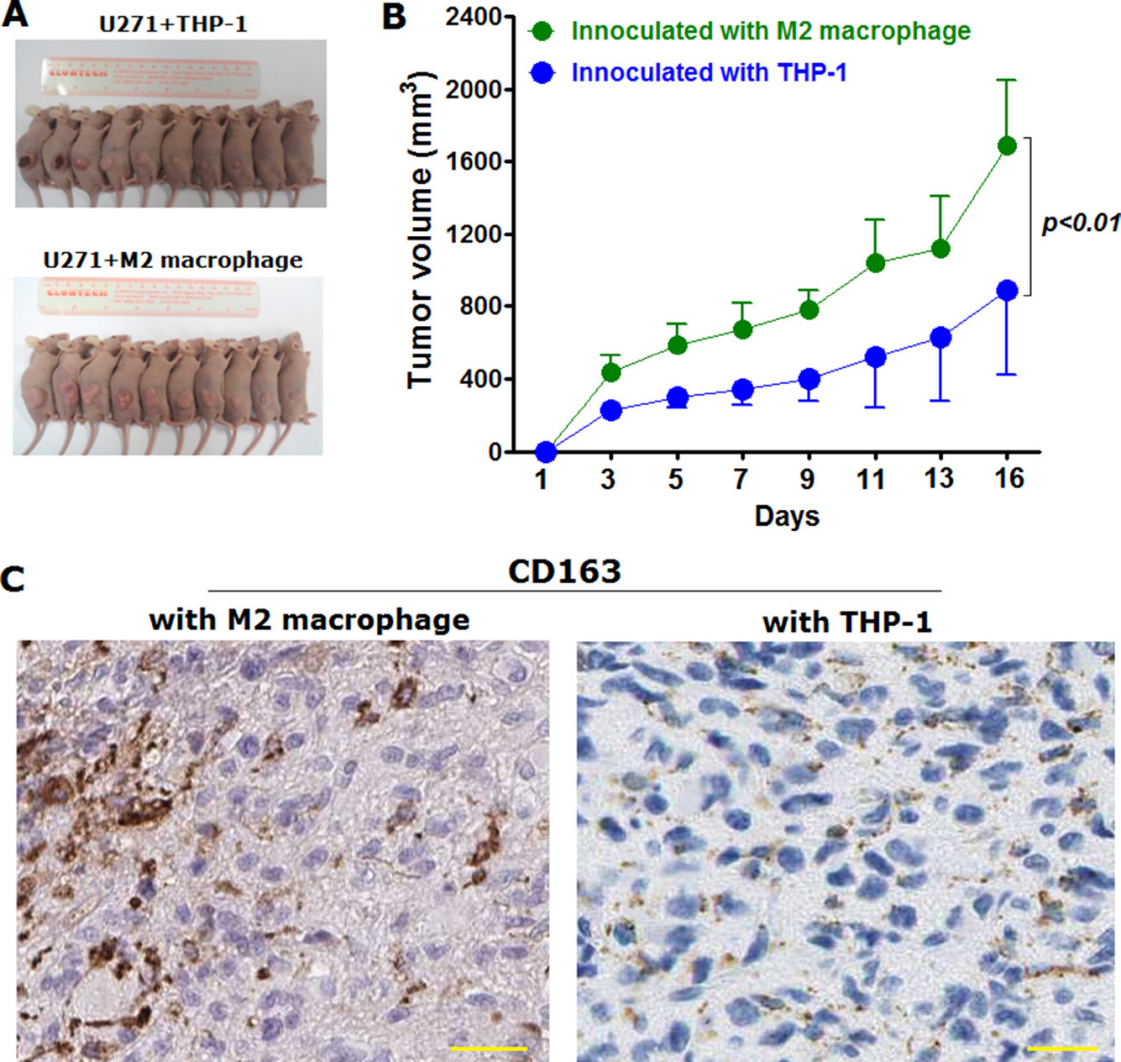
M2 macrophage was displayed to be capable of promoting tumor growth *in vivo* in nude mice xenografted with U271 cells mingled with M2 macrophages or THP-1 cells, respectively (**A**) nude mice were euthanatized at 16th day since subcutaneous inoculation with U271 glioma cells mingled with M2 macrophages and with the same number of THP-1 cells, as control group. Each group has ten male nude mice which were randomly dispersed into two groups; (**B**) tumor volume was statistically analyzed from the tumor lesions dissected from nude mice; (**C**) M2 macrophage was detected using IHC with the immunostaining of CD163 in tumor lesions dissected from the two groups, respectively. Magnification fold was ×400. Two-tailed Independent sample *T* test was employed, extremely statistical significance was regarded at *p* value was less than 0.01 in comparison with control group.

Considering that M2 macrophage was found to be capable of promoting proliferation of glioma cells *in vitro*, we've next determined to go on to confirm *in vivo*. To observe the effects of M2 macrophages over proliferation *in vivo*, we've employed xenografted nude mice model (Figure 4A). Glioma cell lines U271 (2.5 × 10^5^) was subcutaneously injected with the same cell number of THP-1 (2.5 × 10^5^) treated with M-CSF alone or in combination with equal number of M2 macrophages induced (2.5 × 10^5^ U271 mingled with 5.5 × 10^5^ M2 macrophages). The xenografted tumor size and volume were evaluated after being palpable. Furthermore, to make sure THP-1 derived M2 macrophages existed in xenografted tumor lesion, we've verified using IHC with the detection of CD163. It was found that at 16th day after inoculation, the tumor volume of mice subjected to inoculation with U271 mingled with equal number of M2 macrophage were remarkably larger than that from control group where nude mice were inoculated with glioma cells mingled with THP-1 cells (Figure 4B). Moreover, results of IHC detection showed that M2 macrophages were existed in the tumor lesions dissected from nude mice co-inoculation with M2 macrophages, whereas M2 macrophages were hardly detectable in tumor lesions from nude mice co-inoculation with THP-1 cells treated with M-CSF alone (Figure 4C).

### IL-10 from M2 macrophages promoted glioma cell growth through activation of JAK/STAT3 signaling pathway

**Figure 5:**
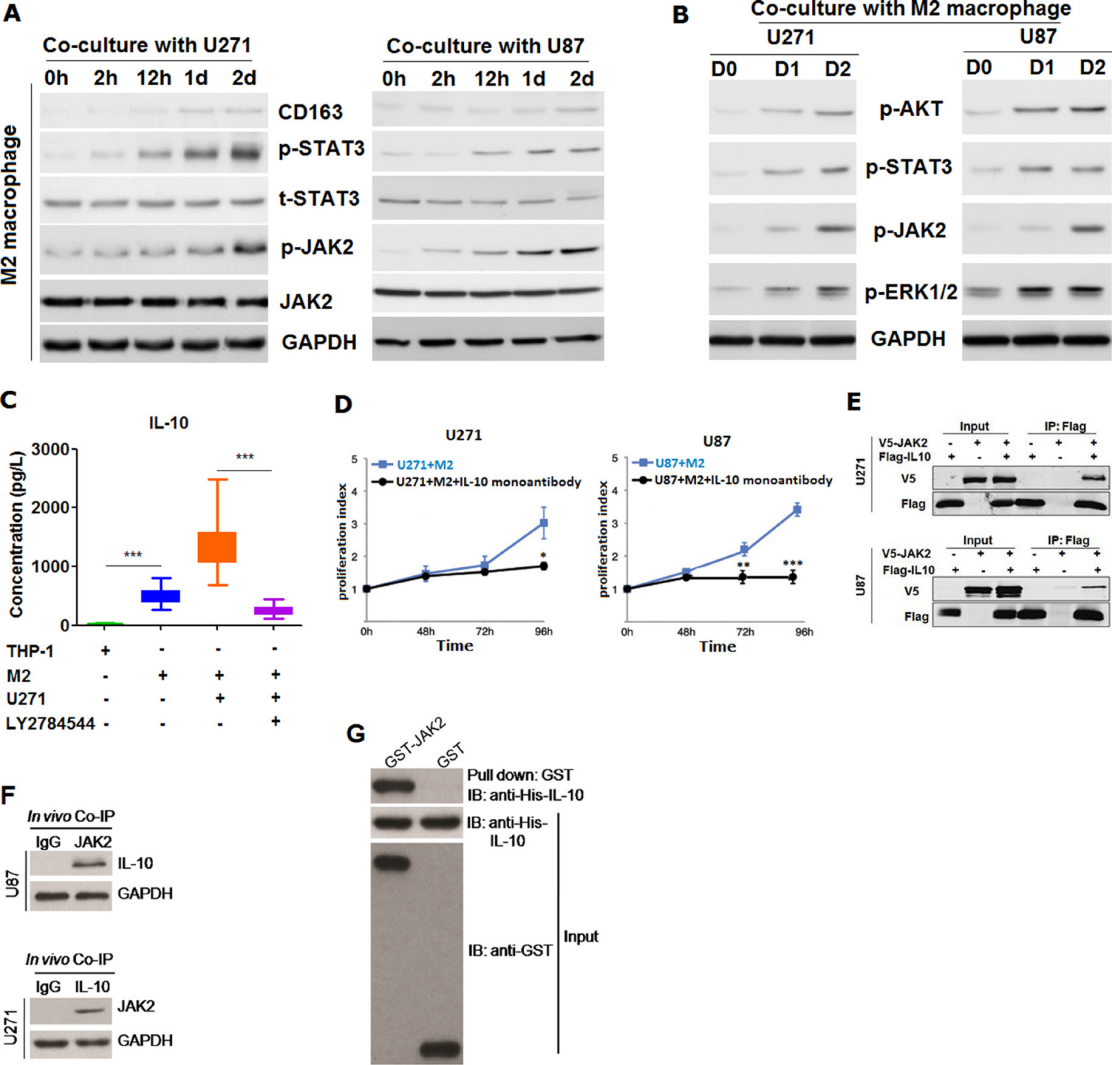
M2 macrophage was found to be able to promote the proliferation of glioma cells through secreting IL-10 dependent on JAK2/STAT3 signaling pathway (**A**) CD163 as well as JAK2/STAT3 signaling pathway were detected using western-blot in THP-1 cell co-cultured without or with U271 and U87 glioma cells for 2hours, 12 hours, 1 day and 2 days. GAPDH was used as internal loading control; (**B**) activated p-AKT, p-STAT3, p-JAK2 and p-ERK1/2 were detected in both U271 and U87 cells co-cultured with M2 macrophage for 0 day, 1 day and 2 days; (**C**) Elevated IL-10 secreted from M2 macrophage co-cultured with glioma cells U271 was remarkably decreased in the presence of 5 μm LY2784544, a kind of specific inhibitor of JAK/STAT3 signaling pathway (Selleckchem, Inc.), as measured by ELISA method; (**D**) enhanced proliferation of U271 and U87 glioma cells by IL-10 secreted from M2 macrophage can be abolished using monoantibody against IL-10, as exemplified by MTT assay; (**E**) IL-10 was found to be able to exogenously interact with JAK2 in a protein-protein interaction, using co-immunoprecipitation (Co-IP) approach; (**F**) shown was IL-10 can endogenously interacted with JAK2 in U271 and U87 cells; (**G**) GST-pull down of JAK2 and IL-10. Two-tailed Independent sample T test was employed in the analysis of statistical difference between two groups, * represents *p* < 0.05, ** stands for *p* < 0.01 and *** means *p* < 0.001 compared with control group. Log-rank test was used in the survival analysis, and differences were considered to be statistically significant at *p* < 0.05.

Having confirmed that M2 macrophage was capable of promoting tumor growth *in vivo*, we've subsequently tried to explore the underlying mechanism by which M2 macrophage played role in the process. First of all, we've detected the expression variation of JAK2/STAT3 signaling pathway in M2 macrophages co-cultured with U271 and U87 cells according to previous relevant report [10]. It was shown that JAK2/STAT3 was gradually activated with co-culture with U271 and U87 cells, which was time-dependent (Figure 5A). Then, we've also measured the p-AKT, p-STAT3, p-JAK2 and p-ERK1/2 in U271 and U87 after co-culture with M2 macrophages induced from THP-1. It was found that both p-AKT, p-STAT3, p-JAK2 and p-ERK1/2 were increasingly activated in U271 and U87 cell after co-culture with M2 macrophage (Figure 5B), indicating that glioma cells U271 and U87 could activate the JAK2/STAT3 signaling pathway in M2 macrophages, which in turn M2 macrophages could also activate both the ERK1/2 and AKT signaling pathway in glioma cells. In the following, on the basis of previously published reports [11–13], we've postulated that there could be existed a cytokine or protein factor secreted by M2 macrophage that contributed to facilitate the malignant behavior of glioma cells. To test our hypothesis, we've analyzed the supernatant of co-culture with M2 macrophage using cytokine microarray. It was found that IL-10 was markedly up-regulated in co-culture media compared with media co-cultured with M2 macrophages relative to co-culture with THP-1 (Supplementary Figure S4A). To further confirm, we've evaluated these strikingly differentiated cytokines screened out by protein microarray using ELISA methods. Results from ELISA were entirely concordant and congruent with that of cytokine microarray (data not shown). Next, we've determined where IL-10 was from. Using ELISA detection, it was shown that IL-10 secreted was pronouncedly higher in the medium of U271 co-cultured with M2 macrophages than that in medium of M2 macrophage cultured alone. Nevertheless, high secretion of IL-10 can be markedly blocked by the addition of LY2784544, one specific inhibitor of JAK2/STAT3 signaling pathway, into the M2 macrophage culture media (Supplementary Figure S4B and Supplementary Figure S5C), suggesting that IL-10 was mainly secreted by M2 macrophages and that secretion of IL-10 from M2 macrophage was dependent on the activation of JAK2/STAT3 signaling pathway (Supplementary Figure S4C and Supplementary S4D). Based on the evidence we obtained, we've reasoned that IL-10 may play a major role in the promoting of proliferation of glioma cells. To test the reasoning, monoantibody against IL-10 was added into the medium of U271 co-cultured with M2 macrophages. We've observed that the promotion of growth was entirely abolished by blocking IL-10 using neutralizing mono-antibody against IL-10 in comparison with control group (Figure 5D).

### IL-10 can interact with JAK2 thereby activating the JAK2/STAT3 pathway

Having found that IL-10 secreted from M2 macrophages was dependent on the JAK2/STAT3 signaling pathway and that co-culture with glioma cells was able to activate the JAK2/STAT3 signaling pathway in M2 macrophages, we've next tried to understand how IL-10 was dependent on JAK2/STAT3 pathway. We've identified that IL-10 can co-operate with JAK2 in a direct protein-protein interaction fashion (Figure 5E, 5F and 5G), suggesting that IL-10 could form complex with JAK2, thereby activating the JAK2/STAT3 pathway. Moreover, considering the pivotal role of IL-10 in the promotion of proliferation of glioma cells, we've meanwhile evaluated IL-10 expression in glioma tissues (Supplementary Figures S4E and S5). It was found that IL-10 was shown to be remarkably up-regulated in glioma tissues compared with paired normal control tissues and that IL-10 was also significantly associated with poorer overall survival (Supplementary Figure S4E) and that up-regulation of IL-10 was also pronouncedly correlated with WHO grade and relapse of patients with glioma (Table 3). Further analysis showed that IL-10 expression can also be used as an independent prognostic factor for patients with glioma (Table 4), suggesting that blocking of IL-10 could be an alternative therapeutic strategy for patients with glioma.

**Table 3:** Analyzed was clinicopathological significance of IL-10 expression in glioma tissues

Variable	group	Total	IL-10 expression		Chi-square	*p* value
			Low (−, +)	High (++, +++)		
Glioma		140	47	93	65.605	0.000
Paired normal control		140	114	26		
Age	< 60	67	27	40	2.607	0.112
	≥ 60	73	20	53		
WHO grade						0.001
	I + II	60	30	30	12.708	
	III + IV	80	17	63		
Gender						
	female	43	12	31	0.893	0.438
	male	97	35	62		
Relapse						
	Yes	105	24	81	21.620	0.000
	No	35	23	12		
Histological subytpe						
	Astrocytic	49	19	30	0.920	0.631
	Oligoastrocytic	45	14	31		
	Oligodendroglial	46	14	32		

**Table 4:** Univariate and multivariate Cox-regression analysis of the IL-10 expression over prognostic parameters in patients with glioma

Clinicopathological parameters	Univariate analysis		Multivariate analysis	
	RR (95% CI)	*p*	RR (95% CI)	*p*
Age (*n* = 140)	0.804 (0.361, 1.791)	0.593		
Relapse (*n* = 140)	3.109 (1.386, 6.974)	0.006	3.286 (1.239, 8.72)	0.017
WHO Grade (*n* =140)	2.977 (0.999, 8.868)	0.05	3.064 (0.863, 10.873)	0.083
Gender (*n* = 140)	1.43 (0.570, 3.588)	0.446		
Histological subtype (*n* = 140)	0.817 (0.342, 1.949)	0.648		
IL-10 immunostaining score	2.695 (1.165, 6.231)	0.02	2.767 (1.061, 7.212)	0.037

## DISCUSSION

M2 macrophage has been reported to be clinically involved in the carcinogenesis of glioma; however, the underlying mechanism has been largely unknown. Here, in our setting, we've found that M2 macrophages were pronouncedly higher in glioma tissues than that in paired normal controls; and that M2 macrophages were significantly and clinically associated with tumor progression and inferior overall prognosis of patients with GBM. Moreover, M2 macrophages were displayed to be able to promote the proliferation and tumorigenesis of glioma cells *in vitro* and *in vivo* through secretion of IL-10 which was dependent on the activation of JAK2/STAT3 signaling pathway, indicating that IL-10, secreted from M2 macrophage, can be targeted in the curing of patients with glioma.

Considering that the current standard therapies, including surgical, radiation and conventional chemotherapy, hardly have had influence over the overall prognosis of patients with glioma, thus, there has been urgent need to find alternative therapeutic strategy. Facing the challenge of standard therapy, immunotherapy is increasingly recognized [2, 4] and appreciated [4]. Given this, there are several potential clinical implications of our study. Firstly, accumulation of M2 macrophage clinically correlates with progression of glioma, strongly supporting therapeutic targeting of M2 macrophage in the curing of glioma [3]. Secondly, we found that tumorigenesis of glioma was dependent on tumor-promoting roles of M2 macrophage; thirdly, IL-10, secreted by M2 macrophage, was shown to be able to promote tumorigenesis and that block of IL-10 can markedly abolish the influence over proliferation of glioma cells exerted by M2 macrophage, demonstrating that IL-10 can be potentially used as therapeutic target in the therapy of glioma.

On account of the frequency of TAMs greatly outnumbers lymphocytes in human gliomas [14], TAMs therefore were thought to play a major role in the promoting of growth of glioma [3, 7]. TAMs were classically categorized into M1 subtype and M2 subtype macrophages. The former was discovered to be tumor-suppressing and the later was observed to be tumor-promoting in gliomas [15]. But the mechanism of M2 macrophage responsible for tumor-promoting remains to be studied. Clinically, the inversed relationship between the number of M2 macrophages and prognosis has been acknowledged by numerous early studies in the setting of glioma [8, 16]. Besides, positive association between M2 macrophage and tumor grade was also extensively found not only in glioma [8, 12, 16] but also extended in other types of cancers. The clinical findings from these earlier reports were wholly in agreement with ours found in the study, indicating that the number of M2 macrophage might be used in the evaluation of tumor grade [17] and prognosis [18] of patients with glioma. In addition, recent studies suggested that M2 macrophage may promote glioma progression in many aspects. For example, cytokines secreted by M2 macrophages, including IL-10 [19, 20], TGF-β [20] and M-CSF [16, 21] have been shown to promote cell migration and invasion in addition to proliferation in the background of glioma cells. Furthermore, M2 macrophages was also discovered to be capable of promoting angiogenesis in GBM through releasing the Insulin-like Growth Factor-binding Protein 1 (IGFBP1) [22], indicating the multiple ways in which M2 macrophage played role in the promoting of GBM. considering that M2 macrophage mainly works in the way of secreting protein factors or cytokines that directly influence over glioma cells, we've screened out the most significant cytokines from M2 macrophage using antibody microarray technique. IL-10 was shown to be most significantly higher among the cytokines secreted by M2 macrophage, which was fully congruent with early reports [19, 20] in glioma. However, despite the authors of both the two early reports have discovered and mentioned that IL-10 was among the cytokines that were remarkably up-regulated and relevant to M2 macrophage, however, the mechanism by which how IL-10 from M2 macrophage played role remains to be investigated in their respective studies. Given this, we've determined to follow up the biological roles of IL-10 from M2 macrophage on the basis of previous publications together with the results obtained on our own.

Mechanistic studies from cell culture system and animal experiments found that proliferation of cancer cells in direct co-culture with M2 macrophages was markedly rapid than that in indirect co-culture system [9, 23], indicating that not only M2 macrophage-produced IL-10 or other soluble factors but also direct cell to cell contact can lead to malignant phenotypes of cancer cells. Besides, direct co-culture with M2 macrophages was observed to be able to pronouncedly activate STAT3 signaling pathway in cancer cells [9, 24], contributing to the proliferation and progression in cancers. With respect to how did IL-10 stimulate the STAT3 signaling pathway remains to be investigated. We showed that it is IL-10 secreted from M2 macrophage that promotes the proliferation of glioma cells, which was in agreement with previous studies. Furthermore, we for the first time found that IL-10 stimulated the JAK2/STAT3 pathway in glioma cell lines U271 and U87 through interaction with JAK2 in a protein-protein interaction fashions. Finally, to confirm the role mediated by IL-10 from M2 macrophage in the promoting of glioma cells, we've blocked the IL-10 using neutralizing mono-antibody against IL-10 both *in vivo* and *in vitro*. We found that the influence over glioma cells exerted by IL-10 can be significantly abolished after blockage of IL-10, relative to control group. Given this, the finding may have clinically translational significance in terms of therapy of glioma. Nevertheless, extrapolation of direct IL-10 function from the study should be approached with caution. Firstly, IL-10 was just shown to be significantly higher in our setting among the interleukins secreted from M2 macrophage, which doesn't mean IL-10 is the only one effective cytokine that played a major role in the mediation of malignant behavior of glioma cells. Secondly, Whether or not blocking of IL-10 could inhibit other signaling pathway mainly involved in the carcinogenesis of glioma remains to be studied. Further basic and clinical investigation may be warranted and needed to evaluate the hypothesis that IL-10 could be druggable in the curing of cancers on systemic as well as cellular level.

In conclusion, our findings suggest that existence of M2 macrophage in glioma tissues was clinically relevant to poorer overall prognosis and tumor progression. Moreover, blockage of the IL-10 secreted from M2 macrophage could be an alternative potential therapeutic strategy in light of the recalcitrance of glioma to the current standard therapeutic strategy.

## MATERIALS AND METHODS

### Clinical tissue samples

The current study got approval from the Medical Ethics Committee of Jilin Medical University. All written informed consent was obtained from each patient involved before undergoing neurogliomectomy. 180 cases of glioma and paired normal control were retrieved and collected from the department of pathology of Jilin Medical University. All cases with hematoxylin-eosin (HE) slides were reviewed and interpreted as glioma by neuropathologists (Zhao DH and Lv P) with experienced expertise in neuropathological diagnosis. All corresponding clinicopathological and clinical history information was retrieved from the hospital information system of the department of Neurology, First Hospital of Jilin Medical University, including demographic, clinical stage, pathological grade, TNM and overall prognosis.

### Cell lines and cell culture

THP-1, human monocyte from peripheral blood, was commercially available from American Type Culture Collection (Rockville, MD, USA) (TIB-202). THP-1 was cultured according to the manufacturer's protocol in RPMI-1640 medium supplemented with 10% fetal bovine serum, penicillin and streptomycin. To induce M2 type macrophages, THP-1 was cultured with monocyte colony stimulating factor (100 ng/mL; Sino Biological Inc. Beijing, China) for 5 days, and were treated with IL-4 (20 ng/mL; Sino Biological Inc. Beijing, China), IL-10 (20 ng/mL; Sino Biological Inc. Beijing, China) and IL-13 (20 ng/mL; Sino Biological Inc. Beijing, China) for 24 hours, followed by confirmation using flow cytometry for detection of CD68 and CD163 expression. We've confirmed that more than 95% of cells after induction mentioned above were CD68+CD163+ by flow cytometry. Two human glioma cell lines U251 and U87 with different pathological grades were obtained from the China Center for Typical Culture Collection. The two glioma cell lines were maintained in DMEM supplemented with 10% fetal bovine serum (FBS, Hyclone) and 100 units/mL penicillin, 100 μg/mL streptomycin at 37°C in a humidified atmosphere of 5% CO_2_.

### Cell proliferation assay

U87 and U251 cells seeded at a density of 1 ×10^5^ cells per well in six-well plates were incubated alone (used as control) or in the presence of a direct co-culture, with the same number of M2 type macrophages. A 1-um pore Boyden chamber (BD, Falcon) were employed for indirect incubation. Cells were counted manually on days 1, 2 and 3 after seeding. After expression had been confirmed only in U87 and U251 cells, not in M2 macrophages, using flow cytometry. The magnetic-activated cell sorting method with microbead-labeled anti-human CD antibody was applied to separate glioma cells U87 and U251 from M2 macrophage in co-culture system.

### Quantitative real-time reverse transcription polymerase chain reaction (qRT-PCR)

Total RNA was isolated using TRIzol^®^ (Invitrogen Inc.) following the manufacturer's instructions. RNA was quantified spectrophotometrically using a Nanodrop 1000 spectrophotometer v3.3 (Thermo Scientific). First-strand cDNA synthesis was then conducted using the cDNA Synthesis Kit (Thermo, USA), according to the manufacturer's instructions. The 20 μL reactions contained approximately 2 μg of RNA and 60 μM oligo (dT) primers. All cDNA samples were then diluted 10-fold before being used in qRT-PCR analyses. Quantitative RT-PCR was performed using the Bio-Rad IQ5 Real-Time PCR System. Each qRT-PCR contained 2 μl of cDNA, 0.5 μl each of forward and reverse primers, and 10 μL of SYBR^®^ Green qPCR Ready Mix^TM^ and double distilled water added to give a total volume of 20 uL. The PCR programming comprised an initial denaturation at 95°C for 30 seconds, followed by 40 cycles of denaturation at 95°C for three seconds and primer annealing and extension at 58°C for 30 seconds. Melt-curve analyses were then conducted before concluding the program with a 4°C hold. All the primers involved in qRT-PCR were listed in Supplementary Table S1.

### Immunohistochemistry (IHC)

Tumor specimens from clinical and mice were fixed in 10% formaldehyde, embedded in paraffin and sectioned into 4 μm thick slices. Sections were deparaffined with xylene and dehydrated with 98% ethanol. Serial sections were stained using standard immunoperoxidase techniques with primary antibodies against CD68 (1:250) and CD163 (1:200). For epitope retrieval, specimens were microwave treated for 20 min before incubation with primary antibodies. Pre-immune IgG serum was used as negative control for immunostaining, and positive staining was visualized with diaminobenzidine, followed by a light counter-staining with hematoxylin. All staining were scored by two separate neurosurgical pathologists blind to the treatment and design protocols, conflicts in the scoring, if any, were resolved by consensus. In terms of expression status, it was generally categorized into high and low expression on the basis of immunostaining intensity. The high expression was defined as moderate (++) and strong (+++) positive immunostaining, whereas the low expression was defined as negative (−) and weak (+) positive, unless otherwise specified. Presented were representative images.

### Western-blot

After treatment, cells were collected and washed with ice cold PBS in twice then lysed the cells with RIPA lysis buffer (20 Mm Tris-Hcl [pH 8], 150 mM NaCl, 0.5% sodium deoxycholate, 5 mM EDTA, 1% Nonidet P-40, 0.1% SDS), supplemented with protease and phosphatase inhibitor cocktails (Sigma Aldrich). Cell lysate were clarified by centrifugation at 12,000 × g for 10 mins at 4°C. The protein concentrations in lysate were measured by using Bradford's method. Equal amount of total protein (50 μg per lane) separated by SDS-PAGE (10 & 15% gels), and transferred into 0.22 μm nitrocellulose membrane (Millipore, USA). The membrane was blocked in blocking buffer (5% non fat milk powder in TBS) for 1 hour in room temperature and then was incubated with primary antibodies overnight at 4°C. After washing the membranes were incubated with the corresponding secondary antibodies included horseradish peroxidase (HRP) conjugated Goat anti-rabbit IgG for 2 hours at 4°C. The signals were visualized using enhanced chemiluminescence (Pierce, Thermal, USA). All the details regarding primary antibodies and secondary antibody were seen in Supplementary Table S2.

### Co-immunoprecipitation (Co-IP)

Glioma cells transfected with a control vector or pCMV-Flag-IL-10 (Origene, USA) were used in this study. The samples were pre-absorbed with 25 μl of protein A/G-Sepharose (50%) for 10 min and immunoprecipitation was performed using 4 μg/ml anti-FLAG at 4°C for 1 hr and then incubated with 30 μl of A/G-Sepharose for an additional hour or overnight. After three washes, the pellets were resuspended in 40 μl of SDS sample buffer and boiled for 5 min. The entire supernatant was subjected to immunoblotting.

### GST-Pull down experiment

Fifty to 50 μg of the GST-JAK2 (full-length) and GST-Tag were washed in the binding buffer (50 mM Tris-HCl, pH 7.5, 0.05% Triton X-100, 300 mM NaCl) and mixed with cell lysates expressing the His-IL-10 binding protein for 1 h at 4°C. The bound proteins were separated from the unbound via washing the beads thrice by centrifugation at 1000 rpm for 1 min. The bound proteins were eluted by boiling for 5 min, followed by the eluent was analyzed by 10% SDS-PAGE and then by immunoblotting and subsequent probing for the His-IL-10 with specific antibody. GST control was included for comparison.

### Quantification of cytokine levels

Levels of chemokines secreted in culture medium were measured using the specific ELISA system kits (Abcam), in accordance with the manufacturer's instructions provided. All the specific ELISA kits regarding chemokines selected were seen in Supplementary Table S2.

### Xenografted nude mice

All animal experiments and operations were strictly in accordance with the protocols given by Animal Ethics Committee of Jilin Medical University. 6 to 8-week-old BALB/c male nude mice were purchased from Charles River Laboratory Animal Technology Co., Ltd. Animal-related experimental procedures were approved by the Institutional Animal Care and Use Committee (IACUC) of Jilin Medical University. All animals were maintained in individual ventilated cages. Twenty mice were randomized into two groups (*n* = 5). One group were subcutaneously injected with U271 glioma cells (2.5 × 10^5^) mixed with the same number of M2 macrophages (2.5 × 10^5^), the other group were injected with the same number of U271 but mixed with THP-1 as the first group did. Tumor volumes were measured by vernier caliper every 2 days. At day 16, all mice were euthanatized and tumor lesions were dissected. Tumor volumes were calculated with the formula: volume = (longest diameter × shortest diameter^2^)/2.

### Statistical analysis

SPSS16.0 software was used for statistical analysis and Graphpad Prism software (5.0 version) was employed to generate all the statistical figures. All datas were presented as mean ± SEM of 3 independent experiments. Student's *t-test* and one-way analysis of variance (ANOVA) were used for cell proliferation, migration and invasion and data on molecular level. The chi-square test and Fisher's extract test were used to analyze the relationship between CD163 expression and clinicopathological information. Kaplan-Meier survival curve was employed to analyze the prognosis, using the log-rank test. Pearson's correlation test was used to evaluate the correlation between mRNA expression of CD163 and chemokines selected in M2 macrophages induced in the study. Statistical difference was considered to be significant at the level of *p value* less than 0.05 in comparison with control group.

## SUPPLEMENTARY MATERIALS TABLES AND FIGURES


