# Supplementary material for: IL-10 secreted by M2 macrophage promoted tumorigenesis through interaction with JAK2 in glioma
Source: Oncotarget. 2016 Sep 28;7(44):71673–85. doi: 10.18632/oncotarget.12317 (PMC5342110; doi:10.18632/oncotarget.12317)
Supplement: Supplementary file 1 [file oncotarget-07-71673-s001.pdf]

IL-10 secreted by M2 macrophage promoted tumorigenesis through interaction with JAK2 in glioma

Supplementary Materials

Supplementary Table S1: Listed were the primers involved when performing qRT-PCR

Gene		sequence from 5′ to 3′
CD163	Forward	TTTGTCAACTTGAGTCCCTTCAC
	Reverse	TCCCGCTACACTTGTTTTCAC
IL-10	Forward	GTCATCGATTTCTTCCCTGTG
	Reverse	ACTCATGGCTTTGTAGATGCCT
MMP-1	Forward	AGCTAGCTCAGGATGACATTGATG
	Reverse	GCCGATGGGCTGGACAG
VEGF-C	Forward	CACGAGCTACCTCAGCAAGA
	Reverse	GCTGCCTGACACTGTGGTA
VEGF-A	Forward	CCTTGCTGCTCTACCTCCAC
	Reverse	ATGATTCTGCCCTCCTCCTT
GAPDH	Forward	ACAACAGCCTCAAGATCATCAGCA
	Reverse	TCATGAGTCCTTCCACGATACCAA

Supplementary Table S2: Listed were the primary antibodies as well as ELISA kits involved in the study

Primary antibody	dilution as working	catalog number	company	clone site	source
CD163	1:1000 for WB<br>1:200 for IHC	ab87099	Abcam		Rabbit polyclonal Ab
CD68	1:250 for IHC	ab53444	Abcam		Rabbit polyclonal Ab
IL-10	1:400 for IHC	ab34843	Abcam		Rabbit polyclonal Ab
IL-10	1:1000 for neutralizing	Ab134742	Abcam		Mice monoantibody
p-STAT3 (Tyr705)	1:1000 for WB	No. 9145	Cell Signaling	D3A7	Rabbit mAb
t-STAT3	1:1000 for WB	No.12640	Cell Signaling	D3Z2G	Rabbit mAb
p-JAK2 (Tyr 1008)	1:1000 for WB	No. 8082	Cell Signaling	D4A8	Rabbit mAb
JAK2	1:1000 for WB	No. 3230	Cell Signaling	D2E12	Rabbit mAb
p-AKT (Ser 473)	1:1000 for WB	No.4060	Cell Signaling	D9E	Rabbit mAb
p-ERK1/2(Thr202/Tyr204)	1:1000 for WB	No.4094	Cell Signaling	D13.14.4E	Rabbit mAb
GAPDH	1:3000 for WB	10494-1-AP	proteintech		Rabbit mAb
Flag	1:2000 for WB	CW0083M	CWBIO, Beijing		Rabbit mAb
V5	1:2000 for WB	CW0095M	CWBIO, Beijing		Rabbit mAb
His	1:2000 for WB	CW0143M	CWBIO, Beijing		Rabbit mAb
GST	1:2000 for WB	CW0085M	CWBIO, Beijing		Rabbit mAb
Secondary antibody					
HRP-Goat anti-rabbit	Ready-to-use for IHC	PV-6000	zhongshanqinqiao		anti-rabbit
HRP-Goat anti-rabbit	1:15000 for WB	ab6721	Abcam		anti-rabbit
IL-10 human ELISA kit		ab100549	Abcam		
MMP-1 human ELISA kit		ab100603	Abcam		
VEGF-C human ELISA kit		ab100664	Abcam		
VEGF-A human ELISA kit		ab119566	Abcam		

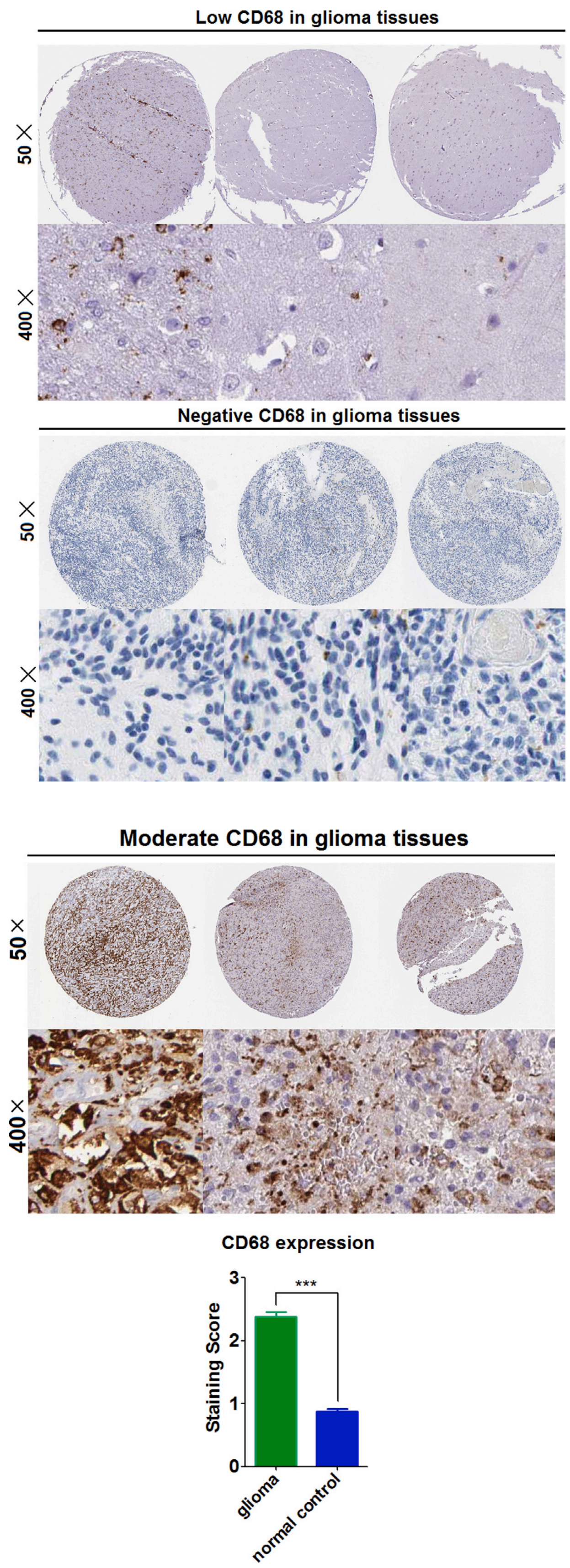

Supplementary Figure S1: Higher expression of CD68 was observed in glioma tissues wherein both CD163 and IL-10 have been detected in parallel in comparison with paired normal control, as shown by IHC. Shown were representative figures of CD68 with no, low and moderate expression in glioma tissues. *** stands for $p < 0.001$ in comparison with paired normal control.

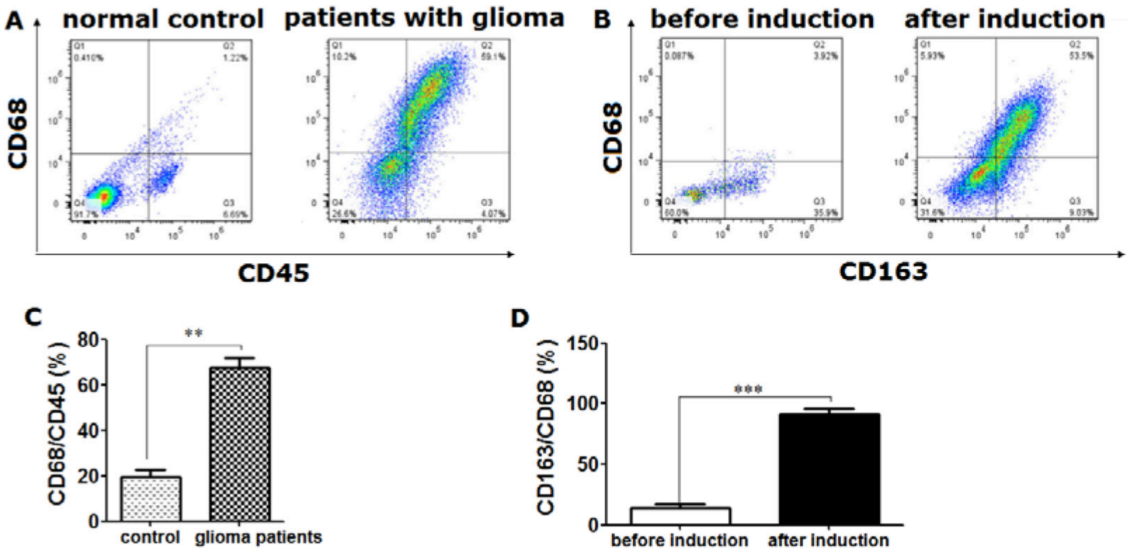

Supplementary Figure S2: (**A**) Shown was the fluorescent-activated cell sorting profiles for a representative case wherein M2 macrophages were analyzed using flow cytometry from peripheral blood of health normal control patient and patient with glioma; (**B**) the purity of M2 macrophages induced from THP-1 was determined by flow cytometry, and representative case was presented here; (**C**) the quantitative assay of 10 cases of M2 macrophage from normal control patients and patients with glioma, as determined by flow cytometry; (**D**) the quantitative assay of purity analysis of M2 macrophage after induction compared with before induction. Three independent flow cytometric analyses were performed. Two tailed independent sample *T* test was employed, and ** represents $p < 0.01$, and *** represents $p < 0.001$ in comparison with control group.

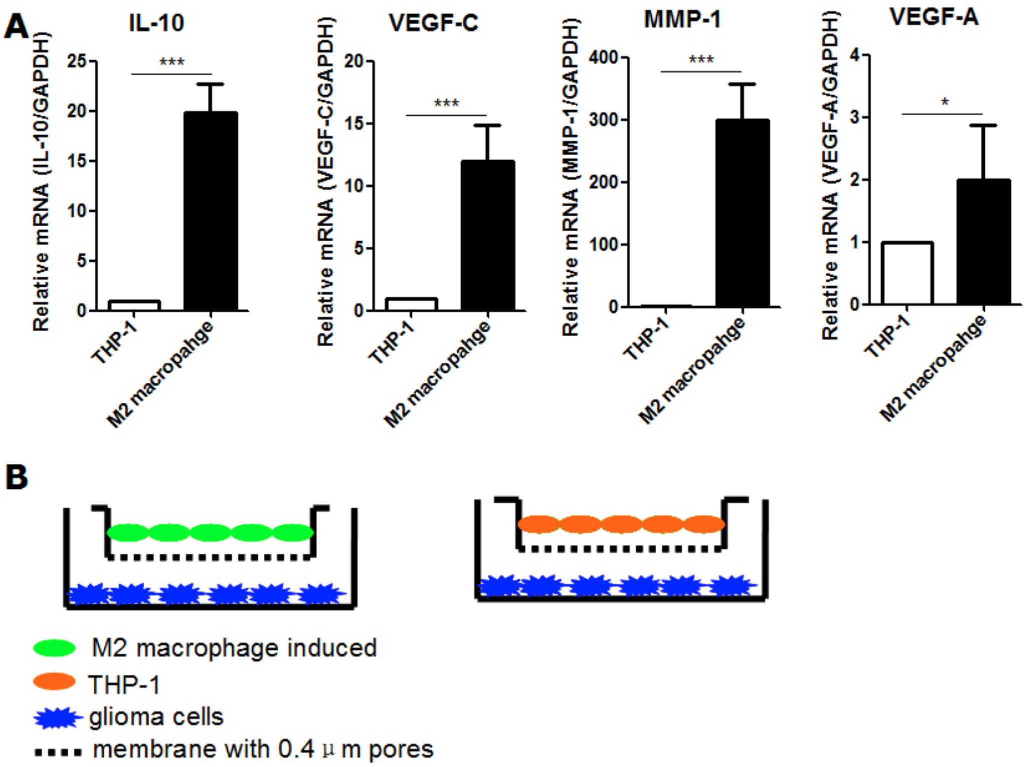

Supplementary Figure S3: (A) Detected was IL-10, VEGF-C, VEGF-A and MMP-1 using qRT-PCR technique in THP-1 cells and M2 macrophages induced from THP-1, as reported previously. Three independent qRT-PCR detections were performed in triplicate; data was expressed as mean plus/minus standard error deviation (STDEV). Two-tailed independent sample *T* test was used in the analysis of statistical significance between two different groups, * stands for $p < 0.05$, ** stands for $p < 0.01$, and *** represents $p < 0.001$ compared with control group; (**B**) co-culture of glioma cells and THP-1 or M2 macrophages using Tranwell culture system in our study. The porous bottom of the insert provides independent access to both sides of a cell monolayer, allowing in vitro cellular interactions.

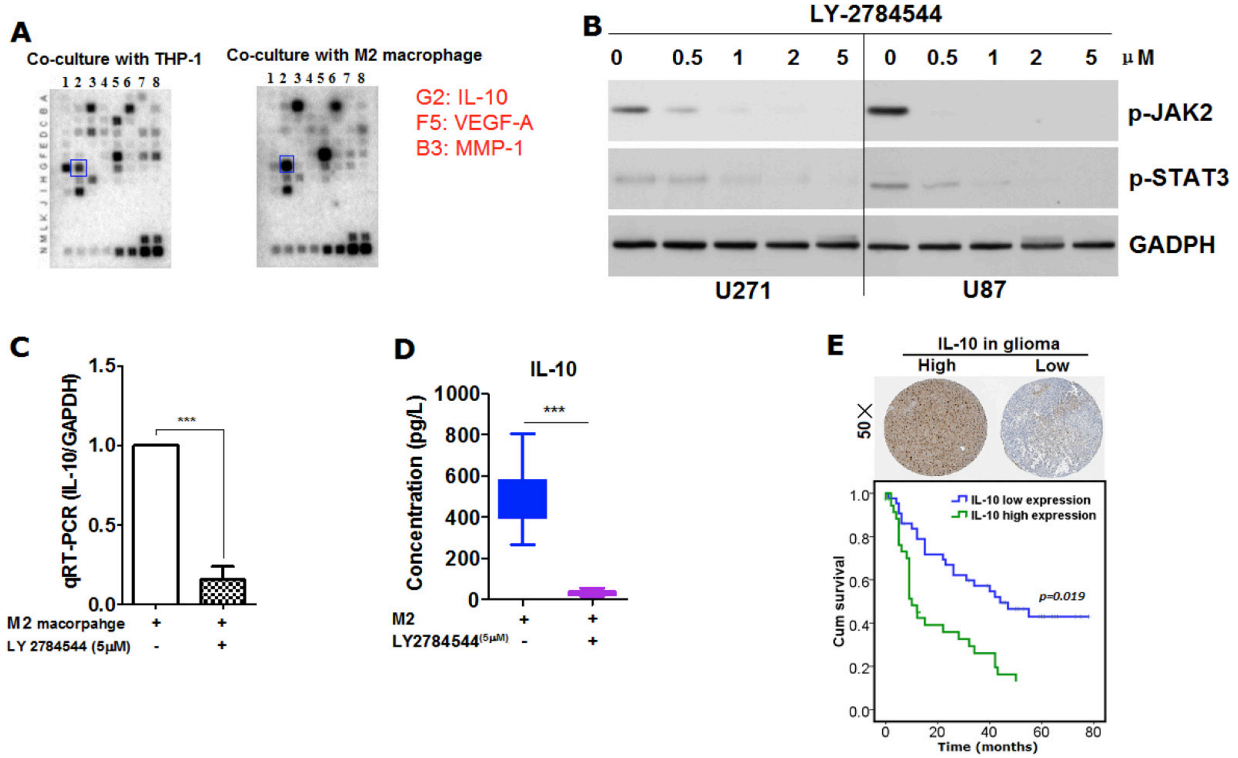

Supplementary Figure S4: Secretion of IL-10 from M2 macrophage was dependent on JAK/STAT3 signaling pathway. (**A**) shown was cytokine array between media of U271 co-cultured with THP-1 and M2 macrophage; (**B**) both p-JAK2 and p-STAT3 can be significantly inhibited in the presence of 5 μm LY-2784544, a kind of specific inhibitor of JAK/STAT3 signaling pathway, for 1 hour in U271 and U87 glioma cells, as exemplified by western-blot; (**C**) mRNA expression of IL-10 was remarkably decreased in M2 macrophages in the presence of treatment of 5 μm LY-2784544 (Selleckchem, Inc.) compared with non-treatment; (**D**) secretion of IL-10 from M2 macrophage was markedly blocked with treatment of 5 μm LY-2784544 in comparison with non-treatment, as measured by ELISA; (**E**) IL-10 was pronouncedly up-regulated in glioma cells whose up-regulation of IL-10 was significantly associated with poorer overall prognosis of patients with glioma. Two-tailed Independent sample T test was employed in the analysis of statistical difference between two groups, * represents $p < 0.05$, ** stands for $p < 0.01$ and *** means $p < 0.001$ compared with control group. Log-rank test was used in the survival analysis, and differences were considered to be statistically significant at $p < 0.05$.

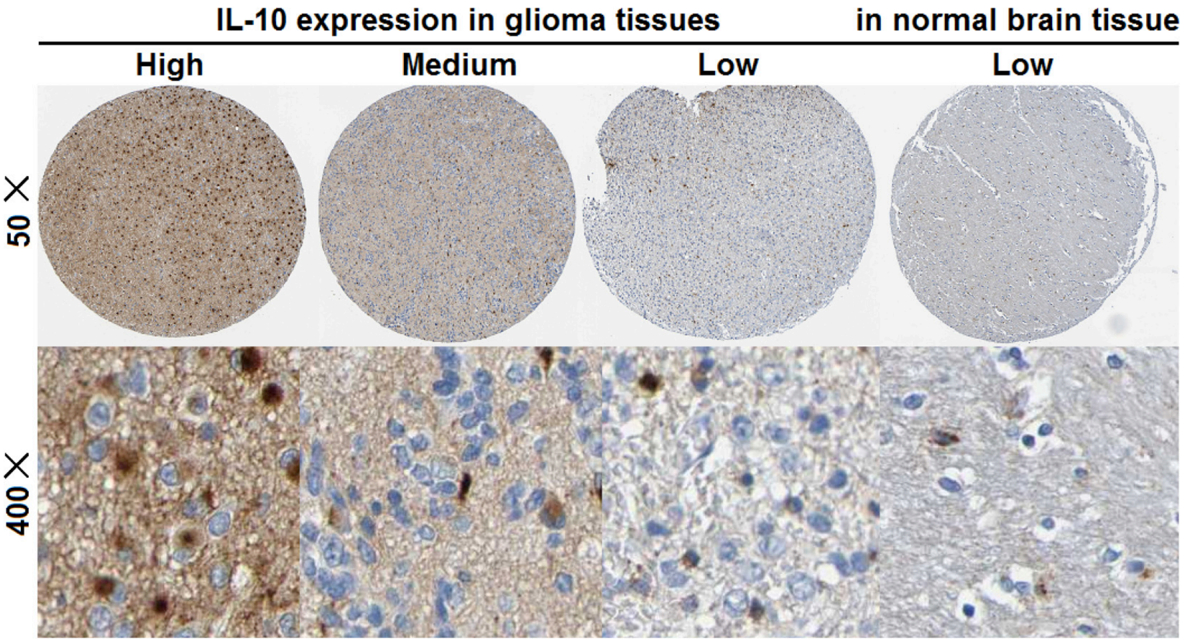

Supplementary Figure S5: Presented was expression status of IL-10 in glioma tissues as well as paired normal control tissue. IL-10 was uniformly positive in glioma tissues wherein both CD163 has been detected previously, with its expression being high, medium and low positive immunostaining in glioma tissues. IL-10 was low or negative in paired normal control tissues.
